# The Role of Cystatin C in the Prediction of Contrast-Induced Acute Kidney Injury Following Coronary Procedures: A Systematic Review

**DOI:** 10.31083/RCM36643

**Published:** 2025-07-29

**Authors:** Azad Mojahedi, Andreas P. Kalogeropoulos, On Chen, Hal Skopicki

**Affiliations:** ^1^Department of Internal Medicine, Stony Brook University Hospital, Stony Brook, NY 11794, USA; ^2^Division of Cardiology, Stony Brook University Hospital, Stony Brook, NY 11794, USA

**Keywords:** acute kidney injury, contrast-induced nephropathy, cystatin C, percutaneous coronary intervention, coronary angiography, systematic review

## Abstract

**Background:**

Contrast-induced acute kidney injury (CI-AKI) represents a significant cause of acute kidney injury (AKI) and accounts for 11% of all cases. Conventional biomarkers, such as serum creatinine (SCr), present limitations in terms of sensitivity and specificity for the early detection of CI-AKI. Therefore, this review examines the potential of cystatin C (CysC) as a biomarker for predicting CI-AKI in patients undergoing coronary procedures and assesses its effectiveness compared to traditional markers.

**Methods:**

This systematic review was conducted using PubMed to identify studies published between January 2020 and March 2025. The inclusion criteria focused on original studies examining CysC levels for early CI-AKI detection in patients undergoing coronary angiography (CAG) or percutaneous coronary intervention (PCI). Data extraction followed a standardized charting method, focusing on key findings from the selected studies.

**Results:**

A total of 7 studies met the inclusion criteria from an initial pool of 410 articles, with data extracted from these seven prospective studies. Key findings indicated that elevated preoperative CysC levels correlated with a higher risk of developing CI-AKI, demonstrating greater sensitivity and specificity than the conventional SCr biomarker. The mean cut-off values for CysC varied across studies, but consistent trends highlighted its potential as an early indicator of renal dysfunction.

**Conclusions:**

CysC appears to be a more sensitive biomarker than SCr for the early detection of CI-AKI. This review suggests that integrating CysC measurement into clinical practice could enhance the early diagnosis and management of CI-AKI, ultimately improving patient outcomes. Hence, future research should focus on standardizing CysC cut-off values and further explore their implications in broader clinical settings and guidelines.

## 1. Introduction

Application of interventional technology in disease assessment and treatment has been extensive. Nevertheless, it frequently results in contrast-induced acute kidney injury (CI-AKI) [1]. CI-AKI is responsible for 11% of all acute kidney injury (AKI) patients [2]. The pathogenesis of CI-AKI, an iatrogenic renal function impairment caused by contrast media, is still obscure. CI-AKI, as an increase of serum creatinine (SCr) ≥0.5 mg/dL (44 µmol/L) or an increase of >25% of baseline within 48–72 h following contrast media exposure, lacks a definitive diagnostic test or therapeutic approach [3, 4]. Combined with appropriate treatments, early clinical diagnosis is necessary to prevent CI-AKI and significantly enhance patients’ prognosis.

Healthcare professionals tend to rely on the assessment of SCr and blood urea nitrogen (BUN) levels in the blood for the diagnosis of CI-AKI [5, 6]. However, it is crucial to note that various internal and external factors like age, gender, diet, weight, and nutritional status influence the level of SCr [7]. In addition, SCr levels may be within normal ranges in most patients irrespective of the risk of CI-AKI following contrast administration and the degree of kidney dysfunction. The limited effectiveness of SCr as a marker of kidney injury was the reason for this. Therefore, it is necessary to determine a biomarker that will predict CI-AKI for the initiation of preventive measures for minimizing the risk of severe kidney damage or failure [6, 7, 8].

Cystatin C (CysC), which inhibits cysteine proteinase, has been investigated as a promising early indicator of renal impairment. The molecule is continuously synthesized by all nucleated cells and released into all body fluids, including plasma, pleural fluid, ascitic fluid, and cerebrospinal fluid. Due to its low molecular weight, CysC freely passes through the glomerular membrane, is entirely metabolized in the kidneys, and is not secreted by the proximal tubules of the kidney [9, 10]. CysC levels were not associated with muscle mass, race, or age. CysC has a half-life of approximately one-third of SCr. Therefore, CysC blood levels stabilize more quickly after renal damage than SCr levels [11, 12]. Earlier research demonstrates that the application of CysC as a predictor of AKI provides earlier identification, perhaps by one–two days, than SCr. CysC also has higher sensitivity and specificity compared to SCr [13, 14, 15]. Therefore, CysC is considered a better marker of renal function than SCr and may become a novel biomarker for AKI in the future.

Coronary angiography (CAG) for diagnostic or percutaneous coronary intervention (PCI) for therapeutic indications, and intra‑arterial use of contrast medium, an iodinated diagnostic contrast agent used for enhancing the blood vessel visualization, which is extensively excreted in the urine in patients with normal renal function, it is extensively excreted in the urine [16]. The standard practice for the detection of CI-AKI is to monitor for a rise in SCr or a fall in urine output [5]. SCr, however, is an imperfect marker for acute renal function impairment. It may not be outside normal limits until renal function has already fallen by more than 50%. Also, SCr levels may be influenced by a variety of factors not related to renal function [11].

Several studies reported the same results. Pre-procedural CysC levels were also demonstrated in a number of other investigations to be an early and valuable biomarker for CAG and contrast scanning of peripheral vascular disease [13, 14]. According to a study, CysC in sepsis patients admitted to the intensive care unit was an early indicator of CI-AKI. Compared with BUN and SCr, which were uninformative, CysC and CysC/Cr ratio had independent predictive value for renal dysfunction following contrast administration. Notably, CysC has been demonstrated to be a promising biomarker for the early diagnosis of CI-AKI [17].

Due to the widespread use of sophisticated surgical methods and risk factor instruments, the majority of research on CI-AKI diagnosis has focused on individuals undergoing CAG or PCI. Hence, this study aimed to determine whether elevated preoperative CysC levels prior to CAG or PCI are associated with a higher risk of CI-AKI occurrence.

## 2. Material and Methods

### 2.1 Search Strategy

We performed a systematic review to examine the utility of CysC level measurement for the early diagnosis of CI-AKI in patients undergoing CAG or PCI. The research was conducted in PubMed, Web of Science, and Scopus from January 2020 to March 2025 using the Advanced Search Builder feature. Search words were used in the [Title OR Abstract] fields. It was limited to original articles in English only, and specific words and phrases and medical subject headings (MeSH) terms for each database were used, for example: ‘(Cystatin C) AND (Acute Kidney Injury OR Nephropathy OR Acute Renal Injury OR Contrast Induced Nephropathy OR Contrast Induced Acute Kidney Injury) AND (Angiography OR Percutaneous Coronary Intervention OR Percutaneous Coronary Revascularization OR Coronary Intervention OR Coronary Revascularization OR Cardiac Catheterization)’.

### 2.2 Inclusion and Exclusion Criteria

This systematic review included original studies that examined the effectiveness of CysC level measurement for early CI-AKI detection in patients undergoing cardiac catheterization, such as CAG or PCI. Studies have reported CI-AKI or contrast induced nephropathy (CIN) as a consequence of the catheterization procedure. Additional relevant literature was identified from the reference lists of the selected studies. Both retrospective and prospective studies, as well as blinded and non-blinded studies, were considered. The exclusion criteria included studies involving participants on renal replacement therapy who had received contrast medium within two days prior to enrollment, had contrast medium allergies, or had undergone aortic valve replacement, renal transplantation, or heart transplantation. Patients with acute heart failure, severe valvular disease, or left ventricular thrombus were also excluded. The review did not include case reports or series with few patients, review articles without original data, editorials, letters, or conference papers.

### 2.3 Data Extraction and Quality Evaluation

Two researchers (AM and OC) examined the titles and abstracts. After applying inclusion and exclusion criteria, relevant information was extracted from the selected studies to meet the survey’s requirements.

Reviewing reference lists from previously published review articles led to the inclusion of pertinent studies. We identified seven eligible research articles in their final published form. We extracted only the principal findings relevant to this review’s scope for specific articles. Data extraction tables compiled from the information from the selected articles are presented in Table 1 (Ref. [14, 18, 19, 20, 21, 22, 23]).

**Table 1.** **Characteristics of the included articles evaluating the measuring CysC levels for early diagnosis of CI-AKI in patients who underwent CAG or PCI**.

Year	Study design	Study population	CI-AKI diagnosis, n (%)	Mean of age, year ± SD (IQR)	Gender, male (%)	Time of measuring CysC	The cut-off point of CysC for predicting CI-AKI	Conclusion	Reference
2024	Case-control study	88	44 (50)	43–69	-	Before and 48 h after PCI	15 ng/mL	This study demonstrated that pre-procedural serum levels of CysC effectively predicted the risk of developing CI-AKI post-procedure.	[23]
2023	Prospective cohort study	300	25 (8.33)	61.03 ± 5.12	176 (58.6)	Before and at 6, 12, 24, 48 h after PCI	A 24 h cut-off value of CysC: 1.08 mg/L	CysC measurement after 24 h has higher diagnostic sensitivity for early CIN prediction than SCr.	[19]
2022	Prospective cohort study	1114	55 (4.94)	66.5–70.5	802 (72)	Before and after CAG^*^	0.92 mg/L	CysC is a sensitive biomarker for early prediction of CI-AKI.	[20]
2022	Prospective cohort study	341	21 (6.16)	58 (52–64)	245 (71.8)	48 h before PCI	1.03 mg/L	According to this study, preoperative CysC levels could predict CIN earlier than SCr, and CysC before PCI was a potential biomarker of renal function following PCI.	[14]
2021	Prospective cohort study	41	6 (14.6)	18–80	32 (40)	Before and 48 h after CAG	-	They discovered that the concentration change of SCr is significantly more effective than CysC as an early biomarker in detecting CIN.	[21]
2021	Prospective cohort study	45	19 (42.2)	66.8 ± 8.2	30 (66.7)	Before and 24 h after CAG	10% increase of CysC level from baseline within 24 h from contrast media exposure	The CysC level measured 24 hours after contrast media exposure more sensitively indicates CI-AKI than the SCr level.	[18]
2020	Prospective cohort study	713	47 (6.7)	66 ± 11	-	24 h before CAG	1.4 mg/L	In 97% of patients, CI-AKI can be ruled out before CAG if the CyC value is less than 1.4 mg/L.	[22]

CAG, coronary angiography; CysC, cystatin C; CI-AKI, contrast-induced acute kidney injury; CIN, contrast induced nephropathy; IQR, interquartile range; PCI, percutaneous coronary intervention; SCr, serum creatinine. * This study does not specify the exact interval for measuring cystatin C, but it states that measurements were taken both before and after the angiographic procedure.

### 2.4 Quality Assessment

Two authors (AM and APK) independently assessed the quality of the published interventions. A third author (HS) resolved any disagreements. To determine the possibility of bias in each of the evaluated studies, the QUADAS-2 instrument was used to evaluate the population, technique, analysis, and reporting requirements of each study [24]. The assessment tool was divided into four key areas: flow and timing, reference standards, index tests, and patient selection. For each individual study, these areas were classified as “low”, “high”, or “unclear”. Following this, classifications across all areas were displayed, accompanied by a subjective evaluation of the overall quality of the studies under consideration.

## 3. Results

### 3.1 Study Selection 

**Fig. 1.**
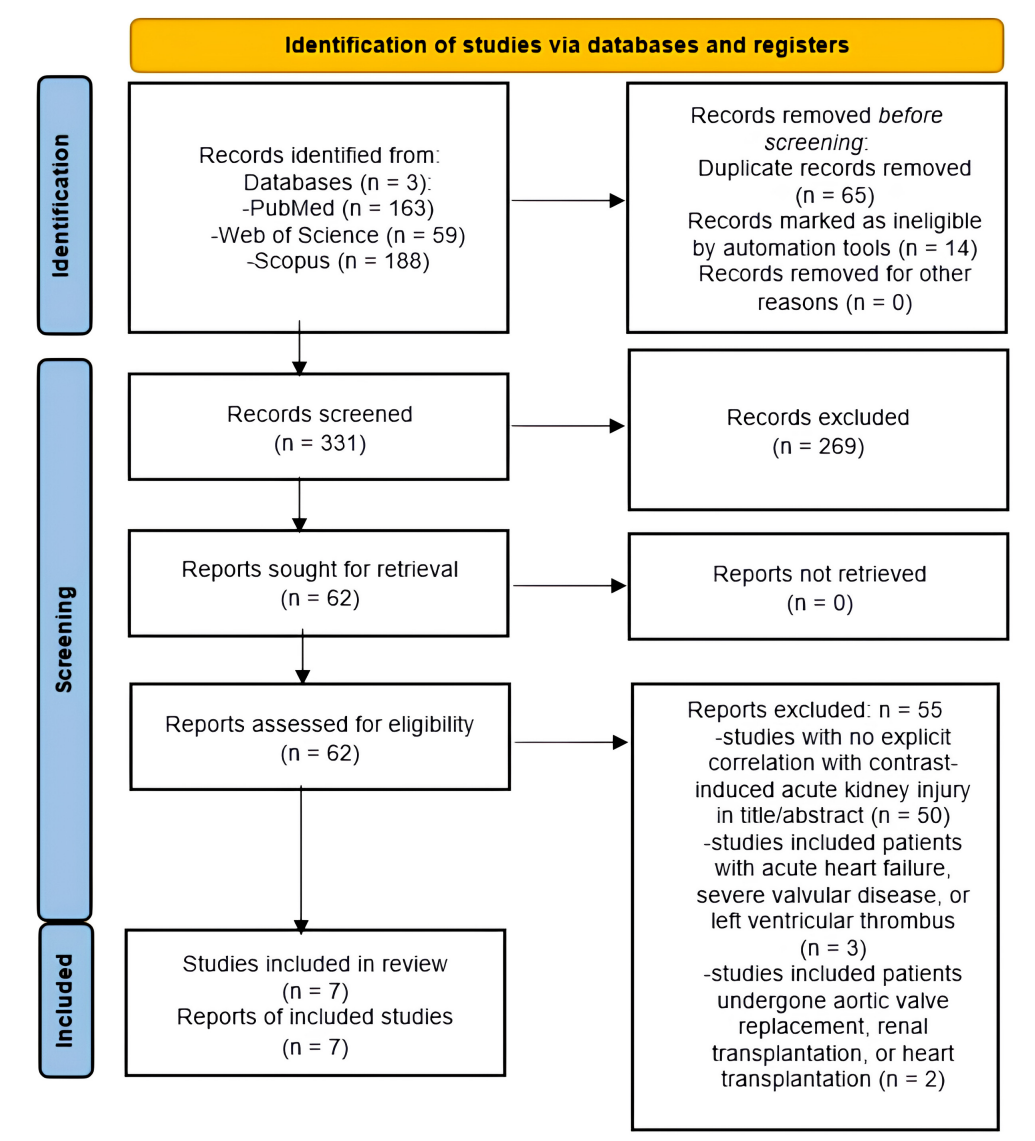
**PRISMA flow diagram for enrollment of studies**.

After conducting a thorough search, we found 410 articles from January 2020 to March 2025. We removed 79 articles, leaving us with 331 studies to screen based on their titles and abstracts. After screening, we excluded 269 studies and were left with 62 to assess their full texts. In total, seven studies met the inclusion criteria for our systematic review. The selection process for these studies is presented in Fig. 1. We extracted data from seven eligible articles, and summarized the information in Table 1.

### 3.2 Quality Assessment

**Fig. 2.**
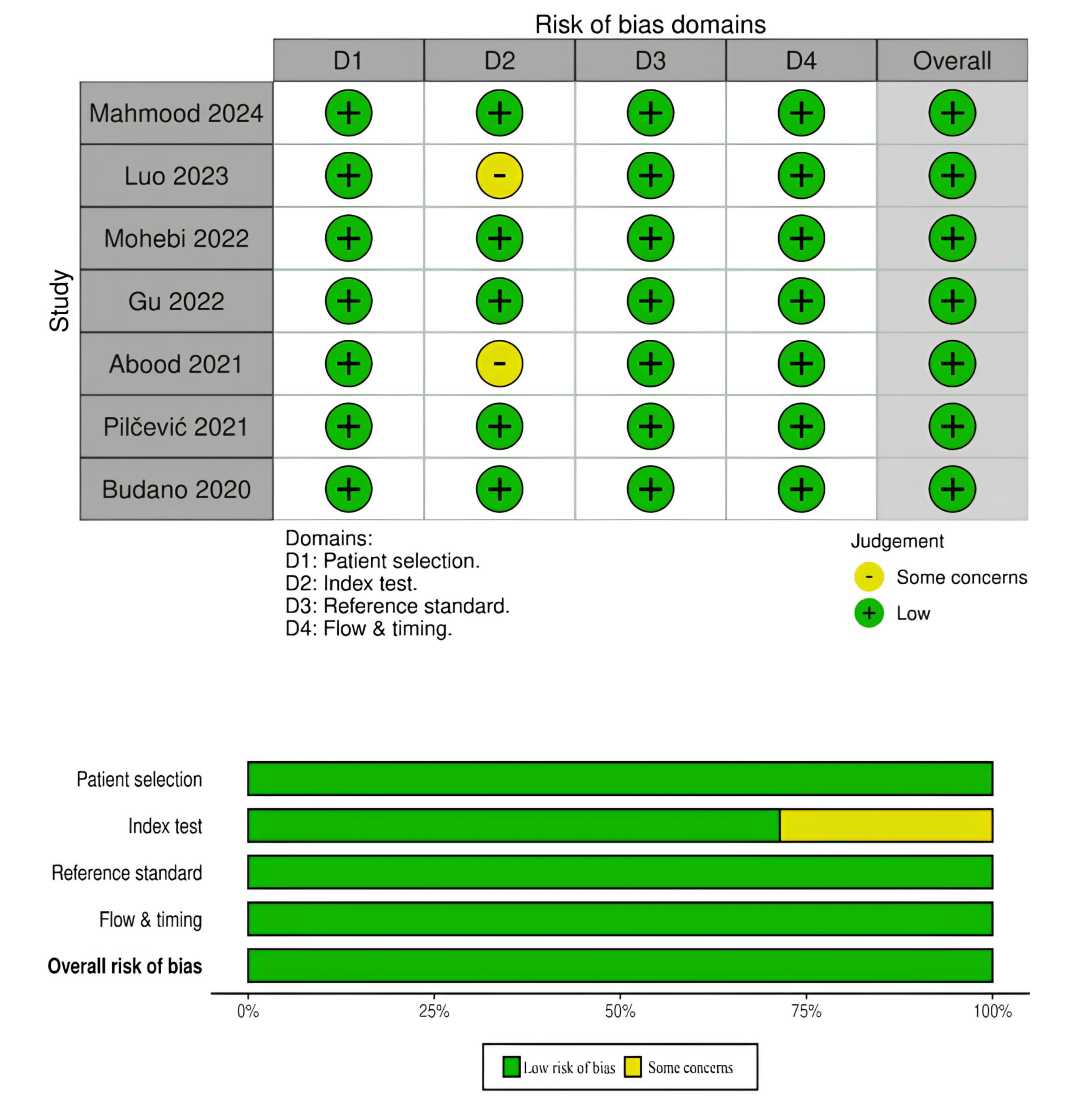
**Quality assessment and bias risk assessment in the investigations included in the systematic review**.

Fig. 2 evaluates the risk of bias across various domains for the evaluated studies. The overall risk of bias of all evaluated investigations were low. However, Luo *et al*. [19] and Abood *et al*. [21] had some concerns regarding the index test domain.

### 3.3 Study Characteristics and Outcomes

The primary population targeted was patients who underwent PCI or CAG. Overall, the evaluated studies had 2642 participants with an age range of 18 to 92 years. Two hundred-four patients developed CI-AKI. There was only one article (Gu *et al*. [14]) in which all participants had normal kidney function. In all studies, at least CysC and SCr were evaluated. Researchers have also assessed various other biomarkers including serum neutrophil gelatinase-associated lipocalin (NGAL), B-type natriuretic peptide (BNP), C-C motif chemokine ligand 14 (CCL14), C-reactive protein (CRP), interleukin-18 (IL-18), osteopontin, and kidney injury molecule 1 (KIM-1). However, due to the aim of our review study, we focus on the level of CysC for early prediction of CI-AKI. All investigations except one evaluated the pre- and post-procedure level of CysC. Budano *et al*. [22] just evaluated the CysC level 24 hours before the procedure.

In 2022, Gu *et al*. [14] presented a study on 341 PCI patients with initially normal renal function. They identified preoperative CysC levels >1.03 mg/L as related to an increased risk of CIN, with an impressive difference in preoperative CysC between CI-AKI and non-CI-AKI groups. In contrast, SCr presented no such distinction, indicating the promise of CysC in detecting renal damage before SCr rises. In 2024, Mahmood *et al*. [23] demonstrated the excellent diagnostic and predictive ability of CysC and CCL14 in 88 patients who were undergoing PCI, with area under the curve (AUC) >0.99 by high sensitivity and specificity using pre-procedure (cut-off 15 ng/mL) and 48-h post-procedure (cut-off 17 ng/mL) values. Similarly, Luo *et al*. [19] found that 24-hour CysC and NGAL post-PCI had slightly higher diagnostic sensitivity (AUC = 0.88) than SCr (AUC = 0.856) in 300 patients. However, sample size limitations and cut-off values were reported. In 2020, Budano *et al*. [22] assessed 713 CAG participants and considered that a single baseline CysC measurement was not only a stronger predictor (AUC = 0.82) of CI-AKI compared to SCr or glomerular filtration rate (GFR) from SCr but also long-term adverse outcomes at 10 years, suggesting a 1.4 mg/L cut-off value that efficiently excluded CI-AKI and pointed towards greater cardiovascular risk. Pilčević *et al*. [18] compared CysC and SCr in 45 patients with chronic kidney disease (CKD) after CAG. They reported that CysC identified CI-AKI significantly more frequently (42.22% vs. 8.89%) at 24 hours, reflecting CysC’s higher sensitivity and proposing a 10% increase in CysC as a reliable early diagnostic marker. However, there is disagreement because another study by Abood *et al*. [21] with 41 patients reported divergent results, showing SCr concentrations significantly increased at 24 hours following contrast in the CIN group but not in the level of CysC. So they concluded that SCr was the better early biomarker in their sample. In summary, all studies except one demonstrated that measuring the CysC level (pre- or post-procedure) was better than SCr for the early identification of CI-AKI. However, the mean cut-off value of the CysC for early detection of CI-AKI was variable in studies.

## 4. Discussion

### 4.1 Overview of CysC

CysC is a protein with a low molecular weight of 122 amino acids and a molecular weight of 13 kDa, garnering increasing attention. It is classified as a cysteine proteinase inhibitor of the type 2 family [25]. Cystine proteases irreversibly break down peptide bonds within amino acid sequences, significantly impacting apoptosis, lipid metabolism, immunological responses, cell control, and cell proliferation and adhesion [9].

The majority of cysteine proteinase inhibitors belong to the superfamily of cystatins. They are widely dispersed throughout animals and allow control over the degradation of extracellular and intracellular proteins. They are, therefore, essential for maintaining the proper ratio of free cysteine proteinases to their inhibitory complexes. Its quantities have been connected to several other illnesses because it enables them to regulate against detrimental cysteine proteinase activities. All nucleated cells synthesize CysC, encoded by the housekeeping *C3T3* gene, at a comparatively steady pace. It is widely dispersed and present in most bodily fluids, including plasma [26, 27].

Due to its unique physical and molecular characteristics, CysC is being considered as a potential kidney function indicator. One of its key features is its constant production rate, which remains unaffected by muscle mass. This, along with its reabsorption and catabolization by proximal renal tubular cells, and its relatively free filtration by glomeruli, makes it a promising candidate for kidney function assessment [28].

### 4.2 The Value of Measuring CysC for the Prediction of CI-AKI in Patients Who Underwent Coronary Intervention

CI-AKI is a serious clinical issue after invasive cardiac interventions, playing a major role in the increased length of hospital stay, high healthcare costs, and negative patient outcomes, such as enhanced morbidity and mortality. Given the substantial impact of contrast media on the incidence of AKI in hospital settings, the clinical significance of CI-AKI warrants considerable attention [29]. The incidence of CI-AKI after primary PCI varies widely, from 4% to 28%, based on the type and amount of contrast medium administered and the particular diagnostic criteria applied. Intermittent intravenous injection of iodinated contrast media during CAG and PCI inevitably increases the risk of nephrotoxicity [30]. With limited universally effective prophylactic measures, detection of CI-AKI, especially in high-risk patient groups, is crucial [15]. Consequently, much research has been directed towards assessing potential early detection biomarkers, among which CysC has been an avid focus of study.

Our systematic review synthesizes robust evidence regarding the clinical utility of pre-procedure CysC concentration as a predictive biomarker of CI-AKI occurrence. In some studies, preoperative CysC levels were better predictors than conventional preoperative SCr levels. Gu *et al*. [14] reported that, among 341 patients with initially normal renal function undergoing PCI, preoperative CysC >1.03 mg/L was strongly associated with an increased risk of CIN. Notably, while baseline SCr values did not differ significantly between patients who developed CIN and those who did not, the corresponding preoperative CysC values were significantly different (*p* < 0.01). This observation suggests that CysC can unveil subclinical renal dysfunction or vulnerability that is not readily detectable by SCr measurements alone. Confirming these observations, Budano *et al*. [22] investigated a larger population (n = 713) undergoing CAG and showed the prognostic value of baseline CysC. Their study established significant validity for CysC in the prediction of CI-AKI, with an AUC of 0.82, which was greater than that for SCr (AUC = 0.70) and SCr-based GFR estimates (AUC = 0.75). Additionally, they demonstrated that a CysC cut-off level of 1.4 mg/L was strongly predictive (97% negative predictive value) for excluding CI-AKI and independently predicted cardiovascular mortality in the long term. Likewise, Mahmood *et al*. [23] observed very high pre-procedural predictive accuracy for CysC with an AUC of 0.999 while studying 88 patients undergoing scheduled elective PCI with baseline normal renal function. The cut-off value suggested by them of 15 ng/mL had a 97.7% sensitivity and 100.0% specificity. Despite the heterogeneity in the analytical methods and cut-off values reported among studies, an evolving consensus is apparent: pre-procedural CysC levels are important for risk stratification prior to contrast media exposure.

Regarding the diagnostic potential of post-procedural CysC levels, several studies suggest that CysC enables earlier detection of renal damage than the traditional marker, SCr. Luo *et al*. [19], in their investigation of 300 post-PCI patients, determined that 24-hour CysC was very accurate diagnostically (AUC = 0.874). This was comparable to NGAL (AUC = 0.885) and better than SCr (AUC = 0.856). In a different group of 45 patients with pre-existing CKD who had undergone CAG, Pilčević *et al*. [18] noted markedly greater incidences of CI-AKI when CI-AKI was defined as CysC increase versus SCr increase at the 24-hour time point (42.22% vs. 8.89%, *p* < 0.001). Based on this discordance, they hypothesized that a modest 10% increase in CysC at 24 h would be an important early diagnostic criterion and that conventional SCr-based definitions are not adequately sensitive in the early post-procedural period. Mahmood *et al*. [23] also provided evidence of the diagnostic usefulness of CysC after the procedure, with the outcome being 48-hour CysC levels that had an AUC of 0.992 for CI-AKI diagnosis. However, this is a later time than that investigated in other studies.

Nonetheless, a definitive consensus regarding the superiority of CysC as an early diagnostic marker remains elusive. Abood *et al*. [21] reported conflicting findings in a smaller investigation involving 41 patients. Their results suggested that the change in SCr level at 24 hours was more indicative of CIN development than the corresponding change in CysC. Specifically, they observed no statistically significant elevation in CysC levels within the CIN subgroup (n = 6) at 24 hours post-contrast administration, whereas SCr levels did increase significantly. This observation challenges the notion that CysC universally rises earlier or more substantially than SCr in all instances of CIN within the initial 24-hour window. The discrepancy between the findings of Abood *et al*. [21] and those of other researchers may potentially be attributable to the limited statistical power inherent in their study, particularly given the very small number of participants who developed CIN, which could constrain the generalizability of their conclusions.

Based on the comprehensive analysis conducted in our systematic review, pre-procedural CysC appears to possess greater efficacy and reliability for predicting CI-AKI risk compared to post-procedural CysC measurements for early diagnosis. The evidence corroborating the predictive value of preoperative CysC exhibits greater consistency across diverse studies and generally demonstrates superior performance relative to traditional SCr measurements for patient risk stratification. While postoperative CysC also shows promise for the early diagnosis of CI-AKI, the findings exhibit greater heterogeneity, with at least one study presenting divergent results. A distinct advantage of measuring CysC preoperatively lies in its capacity to identify individuals at elevated risk before contrast exposure, thereby enabling the potential implementation of targeted preventive strategies in those deemed most vulnerable to CI-AKI.

Overall, CysC emerges as a clinically relevant biomarker for both risk stratification and early diagnosis of CI-AKI following cardiac interventions involving contrast media. Preoperative CysC levels, in particular, demonstrate substantial predictive potential. Future research initiatives should prioritize the establishment of standardized cut-off values, the optimization of measurement timing relative to contrast exposure, and the validation of these findings within larger, more heterogeneous patient cohorts. Such efforts are crucial to enhance and optimize the clinical implementation of CysC assessment in the management of this prevalent iatrogenic complication.

### 4.3 Advantages and Disadvantages of CysC Than SCr for Evaluating Renal Function

SCr level is currently the most commonly examined indicator of renal function. It is released into the bloodstream from the muscle tissue, resulting in a relatively stable rate of entry and individual plasma concentration that varies based on muscle mass, sex, and age. SCr is not attached to plasma proteins, passes freely through the glomeruli, and is minimally reabsorbed by the proximal tubules. However, these tubules secrete small quantities of SCr in the urine. As plasma SCr levels rise, tubular secretion increases, which can lead to an inaccurate overestimation of the GFR in the Rehberg test for patients with moderate to severe reductions in kidney function (<50 mL per minute) [31]. Some studies have shown that CysC-based GFR equations more closely approximate measured GFR compared to SCr-based equations, particularly in certain patient populations like stable kidney transplant recipients [32].

While both SCr and CysC are affected by factors unrelated to GFR, CysC is influenced by fewer variables and to a lesser extent than SCr. SCr levels fluctuate according to factors such as age, sex, muscle mass, exercise, diet, and protein consumption. In contrast, CysC is affected by a different set of non-GFR factors, including systemic inflammation, obesity, thyroid disorders, and steroid use [33, 34].

Additionally, CysC decreases before SCr in patients with AKI, allowing for an earlier prediction of renal recovery. This makes CysC a more sensitive indicator of the early stages of kidney impairment. In patients with heart failure, CysC has shown better predictive value than SCr for adverse outcome prediction. It may identify individuals at higher risk of kidney disease progression than SCr alone would detect [35].

Although CysC provides these benefits, it is crucial to acknowledge that it is not without its drawbacks. As was previously noted, a few factors can affect CysC. Furthermore, the widespread implementation of CysC testing encounters multiple challenges, such as lower familiarity among clinicians and a higher cost than SCr. In practical applications, the most comprehensive evaluation of kidney function may be achieved by combining CysC and SCr, particularly in cases where SCr-based estimates may be suspect. This method can improve the accuracy of estimating GFR and stratifying risk in different clinical settings [9, 12, 36]. Table 2 summarizes the comparison of the CystiC and SCr for the diagnosis of CI-AKI.

**Table 2.** **Summarize the comparison of CysC with SCr to the diagnosis of CI-AKI**.

Characteristic	CysC	SCr
Mechanism	All nucleated cells continuously produce CysC, which inhibits cysteine proteases and is easily filtered by the glomerulus.	The glomerulus filters SCr, which is produced as a result of creatine phosphate metabolism in the muscle tissue.
Advantages	- CysC is less affected by age, sex, race, and muscle mass compared to SCr.	- SCr is widely available and inexpensive.
	- CysC can detect acute kidney injury earlier than SCr.	- SCr has well-established normal ranges and diagnostic thresholds.
Disadvantages	CysC is more expensive and less widely available than SCr.	- SCr is affected by age, sex, race, and muscle mass, which can confound its interpretation.
		- SCr is a less sensitive marker of AKI compared to CysC.
Performance in CI-AKI	- Majority studies have shown CysC can predict CI-AKI earlier than creatinine, with higher sensitivity and specificity.	- SCr is a less sensitive marker for early diagnosis of CI-AKI compared to CysC.
	- CysC levels increase within 12–24 hours after contrast exposure, before SCr.	- SCr may not increase until 48–72 hours after contrast exposure.

AKI, acute kidney injury.

## 5. Strengths and Limitations

This systematic review had several strengths. First, there was considerable consensus among the majority of recent prospective studies in finding elevated CysC as a strong predictor of CI-AKI after coronary interventions. Second, most studies utilized appropriate statistical approaches to assess and quantify the predictive value of CysC, with reported indices such as AUC, sensitivity, specificity, and optimal cut-offs. Third, some studies directly compared the performance of CysC with that of the then-current standard, SCr, and presented helpful comparative data that were favorable to CysC in most instances, particularly for earlier prediction or in patients with an initially standard SCr. Lastly, to our knowledge, no review has specifically examined CysC levels during the period in which the original studies were conducted. However, seven studies were identified between 2020 and March 2025. Thus, the emphasis on research articles over this period in the present systematic review provides a modern assessment in relation to present-day clinical practice with newer contrast media and procedural methods.

Despite these strengths, the evidence base is not without limitations. Several studies were single-centered and observational, potentially restricting generalizability and introducing biases, such as selection and measurement bias. Although CysC assays are now widely available, their cost and availability versus the low cost and ubiquitous availability of the SCr assay still pose practical limitations to widespread use. Furthermore, inconsistencies, such as the iodinated contrast media study, in which CysC levels did not change significantly, highlight areas where the methodology or sample size could have influenced the outcome. Such heterogeneity highlights the necessity for multicenter trials with standardized methodologies to confirm these findings. Moreover, considering that the current study was a systematic review and employed a qualitative approach, it is advisable to conduct a meta-analysis in future research.

## 6. Conclusion

The exact mechanism by which CysC is associated with CI-AKI is not fully understood. Nonetheless, CysC is regarded as a more sensitive indicator of early renal dysfunction than SCr level. It is postulated to provide a more accurate representation of renal tubular function and may detect subtle alterations in kidney performance that SCr measurements alone cannot identify. Therefore, elevated CysC levels may indicate renal tubular damage or dysfunction caused by contrast agents, leading to the development of CI-AKI.

## Availability of Data and Materials

All data generated or analyzed during this study are included in this published article.

## Supplementary Material

Supplementary material associated with this article can be found, in the online version, at https://doi.org/10.31083/RCM36643.
